# Electrically function-switchable magnetic domain-wall memory

**DOI:** 10.1093/nsr/nwad093

**Published:** 2023-04-10

**Authors:** Yu Sheng, Weiyang Wang, Yongcheng Deng, Yang Ji, Houzhi Zheng, Kaiyou Wang

**Affiliations:** State Key Laboratory of Superlattices and Microstructures, Institute of Semiconductors, Chinese Academy of Sciences, Beijing 100083, China; State Key Laboratory of Superlattices and Microstructures, Institute of Semiconductors, Chinese Academy of Sciences, Beijing 100083, China; College of Materials Science and Opto-Electronic Technology, University of Chinese Academy of Sciences, Beijing 100049, China; State Key Laboratory of Superlattices and Microstructures, Institute of Semiconductors, Chinese Academy of Sciences, Beijing 100083, China; State Key Laboratory of Superlattices and Microstructures, Institute of Semiconductors, Chinese Academy of Sciences, Beijing 100083, China; College of Materials Science and Opto-Electronic Technology, University of Chinese Academy of Sciences, Beijing 100049, China; State Key Laboratory of Superlattices and Microstructures, Institute of Semiconductors, Chinese Academy of Sciences, Beijing 100083, China; College of Materials Science and Opto-Electronic Technology, University of Chinese Academy of Sciences, Beijing 100049, China; State Key Laboratory of Superlattices and Microstructures, Institute of Semiconductors, Chinese Academy of Sciences, Beijing 100083, China; College of Materials Science and Opto-Electronic Technology, University of Chinese Academy of Sciences, Beijing 100049, China

**Keywords:** spintronics, spin orbit torques, magnetic domain-wall, SOT-MRAM, information security

## Abstract

Versatile memory is strongly desired for end users, to protect their information in the information era. In particular, bit-level switchable memory that can be switched from rewritable to read-only function would allow end users to prevent important data being tampered with. However, no such switchable memory has been reported. We demonstrate that the rewritable function can be converted into read-only function by applying a sufficiently large current pulse in a U-shaped domain-wall memory, which comprises an asymmetric Pt/Co/Ru/AlO_x_ heterostructure with strong Dzyaloshinskii-Moriya interaction. Wafer-scale switchable magnetic domain-wall memory arrays on 4-inch Si/SiO_2_ substrate are demonstrated. Furthermore, we confirm that the information can be stored in rewritable or read-only states at bit level according to the security needs of end users. Our work not only provides a solution for personal confidential data, but also paves the way for developing multifunctional spintronic devices.

## Teaser

Wafer-scale magnetic domain-wall memory arrays can be electrically switched between rewritable and read-only states at bit-level, according to the security-needs of end-users.

## INTRODUCTION

With the unprecedented expansion of information technology, the large amount of data produced by an abundance of terminals calls for memory with increased versatility, advantageous performance and high security, which is in particular demand by end users. Complementary-metal-oxide-semiconductor (CMOS) based memory, as the current mainstream memory, is the main bottleneck of computer systems [1,2]. Researchers have made great efforts with regards to creating versatile memory with high-performance and low-power operation, producing, for example, resistance change memory [3–6], phase change memory [7–10], ferroelectric memory [11–14] and spintronic memory [15–19]. All these reported memories so far only have a rewritable function, and thus the stored information is at risk of being tampered with by hackers through the internet, and this cannot be fully resolved by encryption. Read-only memory can effectively protect data integrity, but existing memory solutions do not allow end users to selectively set the important data to read-only at bit level.

Domain-walls driven by spin orbit torques (SOTs) have a reciprocating motion, meaning they have great potential in the area of rewritable memory due to their non-volatility, high speed and low power consumption [17,18]. If the domain-wall can be electrically annihilated, the device can be turned into read-only. However, the high potential step caused by exchange bias makes the domain-wall hard to cross and annihilate with a current without damaging the device [20]. To realize function-switchable memory, the height of the potential step in different regions of the U-shaped device should be carefully designed to guarantee the device works in both the rewritable and read-only functions.

Here we report on the experimental realization of wafer-scale domain-wall memory arrays for high performance and high security. This U-shaped domain-wall memory, composed of a Pt/Co/Ru/AlO_x_ heterostructure, can be electrically switched from rewritable to read-only function at bit level. With the strong Dzyaloshinskii-Moriya interaction (DMI) of the asymmetric multilayer and reduction of critical current by controlling the deposition of the AlO_x_ layer [21], the rewritable function can be realized by field-free current-induced domain-wall reciprocating motion by SOTs. The stored information can be converted to a read-only state bit by bit through domain-wall annihilation at the edge of the ferromagnetic layer after applying a sufficiently large current. Thus, the read-only bits become immune to electric current pulses, ensuring information integrity when under threat from hackers or impostors.

## RESULTS

### Electrical operation of a U-shaped SOT-driven domain-wall memory device

**Figure 1.**
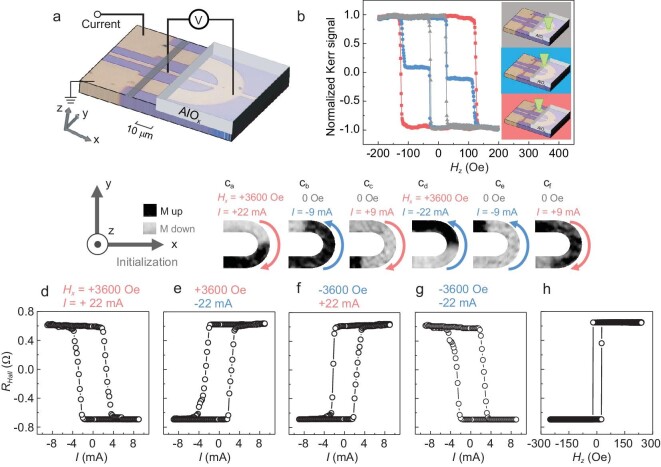
Device structure, magnetization switching by magnetic field, and purely current-driven domain-wall motion. (a) Optical image of the U-shaped device and anomalous Hall effect measurement configuration with the definition of x–y–z coordinates. The gray stripe at both ends of the U-shaped region are trenches where the Co/Ru bilayer is etched away by the argon ion beam. (b) Out-of-plane magnetic hysteresis loops, obtained by a polar magneto-optical Kerr effect (pMOKE) microscope, of the U-shaped device in the Pt(4 nm)/Co(0.85 nm)/Ru(1.2 nm) region (red), Pt/Co/Ru/AlO_x_ (0.5 nm) region (gray) and the boundary (blue), as shown in the insets. (c_a_–c_c_) pMOKE images of the magnetic domain states with initialization of *H_x_* = 3600 Oe and *I_pulse_* = +22 mA (c_a_), and images after *I_pulse_* = −9 mA(c_b_)/+9 mA(c_c_) in the absence of *H_x_*. (c_d_–c_f_) pMOKE images with initialization of *H_x_* = 3600 Oe and *I_pulse_* = −22 mA (c_d_), and after *I_pulse_* = −9 mA(c_e_)/+9 mA(c_f_) in the absence of *H_x_*. Light- and dark-gray regions indicate magnetization-down and -up domains, respectively. (d–g) Field-free current-induced deterministic switching loops of perpendicular magnetization in the curved section (AlO_x_ capped) for four types of initializations: *H_x_* = ±3600 Oe and *I_pulse_* = ±22 mA. (h) Out-of-plane magnetic-field-induced magnetization switching loop in the curved section.

To effectively create and control the magnetic domain-wall by SOTs, a U-shaped device was designed consisting of asymmetric Pt(4 nm)/Co(0.85 nm)/Ru(1.2 nm) multilayers. An AlO_x_(0.5 nm) capped layer was deposited on the curved region to locally weaken the perpendicular magnetic anisotropy (PMA) through tuning the density of defects by ion irradiation [21] (Fig. 1a). Magnetic hysteresis loops were measured using a polar magneto-optical Kerr effect (pMOKE) microscope, with a focused laser spot positioned at the straight section, curved section and the boundary between them (Fig. 1b). The switching fields of the curved and straight sections are 25 Oe and 125 Oe, respectively, confirming the role of the AlO_x_ capped layer. Double-step switching of the hysteresis loop reveals that the domain-wall can be pinned at the boundary of the straight and curved sections (Fig. 1b). The design of the PMA difference is to constrain the domain-wall motion in the low PMA region under a small current in the rewritable function, and also allow the domain-wall to cross over the boundary under a relatively large current intensity without damaging the device during function switching.

Theoretically, the z-component of SOTs effective field is given by [22],


(1) \begin{eqnarray*}
H_z^{\textit{SOT}} = \frac{\hbar }{{2e{M}_st}}\ {\theta }_{\textit{SH}}{J}_x{m}_x ,
\end{eqnarray*}

where $\hbar $ is the reduced Plank constant, *e* is the electron charge, ${M}_s$ is the saturation magnetization, *t* is the thickness of the ferromagnetic layer, ${\theta }_{SH}$ is the spin Hall angle of heavy metal, ${m}_x$ is the x-component of magnetization orientation and ${J}_x$ is the x-component of charge current density. The $H_z^{\textit{SOT}}$ direction is defined by both directions of ${J}_x$ and ${m}_x$. In the U-shaped device, the ${J}_x$ changes its direction as it passes the centerline of the curved section. Therefore, the $H_z^{\textit{SOT}}$ for the up- and down-regions of the device will be opposite with the same direction of ${m}_x$ consistently determined by an in-plane magnetic field, leading to opposite domains formed at both sides of the centerline of the curved section, which is confirmed by the pMOKE images in Fig. 1c_a_ and c_d_. The up-region prefers downward magnetization and the down-region prefers upward magnetization after initialization with *H_x_* = 3600 Oe and *I_x_*= 22 mA, where the duration of current pulses is set to be 10 ms for this paper except where stated (Fig. 1c_a_). But opposite magnetic domain configurations are formed after *H_x_* = 3600 Oe and *I_x_*= −22 mA (Fig. 1c_d_). If both the magnetic field and current directions are reversed for initializations, the same magnetic domain configurations are formed. By constructing asymmetric stacks of Pt/Co/Ru with large DMI, the left-hand Néel domain-wall (⊙$\vert\bigcirc\times$: ↑$\leftarrow $↓ or $\bigcirc\times \vert$⊙: ↓→↑) can be stabilized [23,24]. Thus, the electric current can move the domain-wall along the current direction without the assistance of an external magnetic field.

We then investigated field-free current-induced magnetization switching with the domain-wall motion using both the anomalous Hall effect and pMOKE for different initialized magnetic domain configurations. As shown in Fig. 1d, after the initialization with *H_x_* = 3600 Oe and *I_pulse_* = 22 mA, a clockwise current-induced switching loop at zero field was observed, where the current pulses scanned from +9 to -9 mA, and then back to +9 mA with each point representing a single pulse. The positive current favors the domain-wall motion from up-region to down-region, while the negative current favors the opposite domain-wall motion, resulting in the magnetization downward and upward, respectively. However, after the initialization with *H_x_* = 3600 Oe and *I_x_*= −22 mA, anticlockwise current-induced magnetization switching was observed (Fig. 1e). As expected, for the same initialization of the magnetic domain configurations, the device showed the same deterministic current-induced magnetization switching (Fig. 1d and f, and Fig. 1e and g). The switching magnitudes of *R_Hall_* after initializations of *H_x_* = 3600 Oe and *I_x_*= 22 mA, *H_x_* = 3600 Oe and *I_x_*= −22 mA, *H_x_* = −3600 Oe and *I_x_*= 22 mA, and *H_x_* = −3600 Oe and *I_x_*= −22 mA are 1.30 Ω, 1.31 Ω, 1.31 Ω and 1.30 Ω, respectively, which are very close to the switching amplitude of 1.34 Ω (∼97%) by the magnetic field (Fig. 1h). The critical switching current densities for these four initialized configurations are (2.5 ± 0.2) × 10^6^ A/cm^2^, indicating low power consumption in application. By driving the domain-wall with microsecond short pulses, we found that the motion speed of the domain-wall is at least 14.5 m/s, so that the function speed can be improved to an ns-regime by shrinking the device to nm-scale. In contrast, the referenced symmetrically stacked Pt/Co/Pt U-shaped devices (Fig. S1) have much smaller switching amplitude (12%–35%) and the current-induced switching direction is uncorrelated with the initialization [25,26], indicating the importance of the large DMI for SOT-based domain-wall devices.

### Narrow resistance distribution and high reproducibility

**Figure 2.**
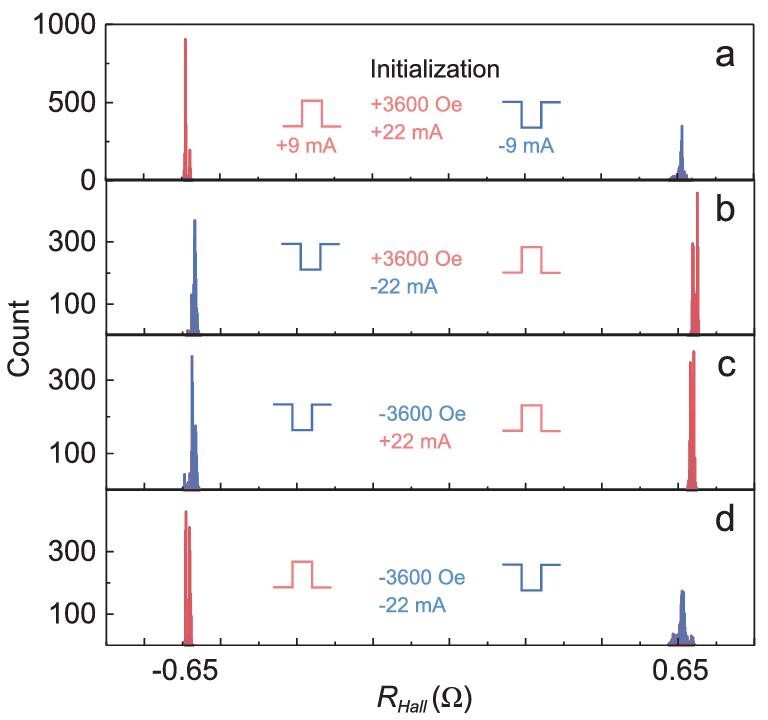
Narrow resistance distribution under alternately positive and negative current pulses. (a–d) Histograms of resistance distribution with a bin size of 0.0015 Ω under alternating current pulses for all types of initializations: *H_x_* = ±3600 Oe and *I_pulse_* = ±22 mA. The applied pulse sequences are alternately positive and negative current pulses with an amplitude of 9 mA and duration of 10 ms for 2000 cycles.

To achieve error-free rewritable domain-wall memory and robust read-out, the average change in resistance (ΔR) from the low to high state should be at least 12σ to have working memories with bit counts of Mb or more [27], where the standard deviation $\sigma \ = \sqrt {\mathop \sum \nolimits_{i = 1}^n {{( {{R}_i - {R}_{ave}} )}}^2/n} \ $, ${R}_{ave}$ is the averaged anomalous Hall resistance ${R}_i$ of each state, and n = 2000 is the total number of pulse cycles. For all four types of initializations, we realized the rewritable function with high reproducibility using alternating current pulses (Fig. 2a–d). The ratios of ΔR/σ for each type of initialization, *H_x_* = 3600 Oe and *I_x_*= 22 mA, *H_x_* = 3600 Oe and *I_x_*= −22 mA, *H_x_* = −3600 Oe and *I_x_*= 22 mA, and *H_x_* = −3600 Oe and *I_x_*= −22 mA, are 177, 240, 223 and 146 respectively, proving that the device shows excellent error-free switching and read-out ability. A wide current-induced switching window up to 10 mA with ΔR/σ higher than 12 is obtained for initialization of *H_x_* = 3600 Oe and *I_x_*= 22 mA (Fig. S2), ensuring stable switching for applications.

### Function switching in a domain-wall memory device

**Figure 3.**
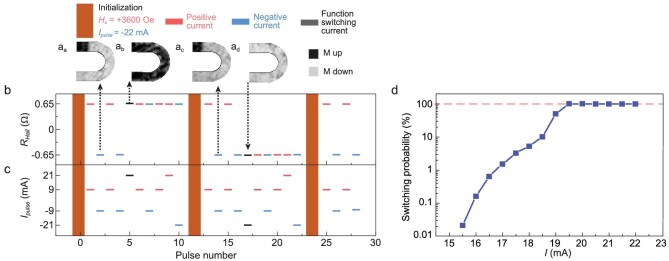
Function switching of the domain-wall device. (a–c) The measured magnetic domain-wall configurations (a_a_–a_d_) and *R_Hall_* (b) after each current pulse (c). Red (blue)-line segments indicate the positive (negative) current pulses and the corresponding *R_Hall_* after the current pulse, and the black-line segments indicate the current pulse for function switching from rewritable to read-only and the corresponding *R_Hall_*. The orange area represents initialization with *H_x_* = 3600 Oe and *I_x_*= −22 mA. Insets show pMOKE images of the corresponding magnetic states indicated by the dashed arrows (a_a_–a_d_). The pulse durations are all 10 ms. (d) The switching probability from rewriteable to read-only function as a function of the current pulse amplitude.

To convert the device from rewritable to read-only function, a large current pulse is needed to annihilate the domain-wall. After initialization of *H_x_* = 3600 Oe and *I_x_*= −22 mA (Fig. 3), the +/−9 mA current pulses can drive the domain-wall back and forth to realize the rewritable function at zero magnetic field. Then, a large current pulse of +21 mA is injected into the device, and *R_Hall_* switched from a low-resistance state to a high-resistance state. After that, the *R_Hall_* remains in the high-resistance state and cannot be changed even with very large current pulses of +/−21 mA (Fig. 3). Similarly, a −21 mA current pulse led to a low-resistance state in the device. Therefore, after a large current pulse was applied, the device entered the read-only function associated with the annihilation of the domain-wall, as confirmed by the pMOKE images in Fig. 3a_b_ and a_d_. We further studied the dependence of switching probability from rewriteable to read-only function on the current pulse magnitude. The switching probability was <0.01% with a current smaller than 15.5 mA, increasing to 100% with a current of 19.5 mA and above (Fig. 3d and Fig. S4).

Interestingly, with the assistance of an in-plane magnetic field, the device in read-only function was electrically reconverted to rewritable function along with the regeneration of the domain-wall (Fig. 3c).

### Demonstration of wafer-scale function-switchable domain-wall memory arrays

**Figure 4.**
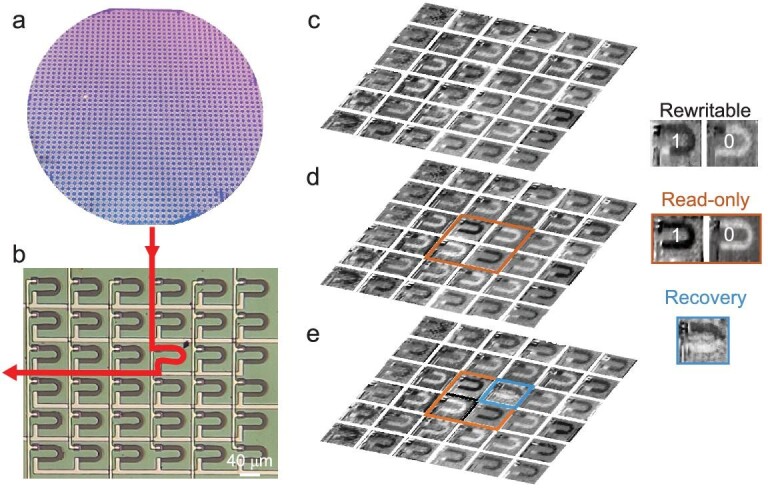
Wafer-scale function-switchable domain-wall memory arrays. (a) Optical image of the function-switchable magnetic domain-wall memory arrays fabricated on a 4-inch silicon wafer. (b) Optical image of the zoomed-in 6 × 6 array of the wafer-scale devices, where the red line with arrows indicates the path to inject a current pulse to a single device. (c–e) pMOKE images of the zoomed-in 6 × 6 array. The right column defines the pMOKE images for ‘0’ and ‘1’ in rewritable and read-only state, and also recovery state. (c) Under the rewritable function, a selected set of data ‘0’ and ‘1’ was written. (d) Four bits in the center of the array were converted to read-only function of ‘1’ (‘0’) by a current pulse of +(−)21 mA, as shown in the brown frame. (e) One read-only bit was reversed back to rewritable function by a combination of an in-plane magnetic field of 3600 Oe and a current pulse of −22 mA (brown frame changing to blue frame).

We fabricated function-switchable domain-wall memories on a 4-inch Si/SiO_2_ substrate (Fig. 4a) using a standard CMOS-compatible process. The optical image of the zoomed-in 6 × 6 array of the wafer-scale devices is shown in Fig. 4b. Each device in the array can be electrically controlled by selecting the corresponding row and column electrodes. The information in our function-switchable domain-wall memory can be stored in rewritable state or read-only state at bit level according to the demands of end users. A set of data in rewritable function was written by current pulses of +/−9 mA. As shown in Fig. 4c, the bits in the outermost circle are ‘1’, written using +9 mA (magnetization downward), the bits in the middle circle are ‘0’, written using −9 mA (magnetization upward), and the 2 × 2 bits in the center are ‘1’, written using +9 mA as well. The 2 × 2 bits in the center were converted into read-only function of ‘1’ using a current pulse of +21 mA and ‘0’ using a current pulse of −21 mA (Fig. 4d, brown frames). The read-only state cannot be changed solely by electrical current since it has no domain-wall (Fig. 4d), thus eliminating the possibility of data tampering by hackers through the internet. With the aid of an in-plane magnetic field, the read-only bit cell can be electrically converted back to a rewritable cell without affecting other bits (Fig. 4e, blue frame). To our knowledge, such recoverability is not available with conventional read-only memory. The wafer-scale switchable domain-wall memory not only allows end users to store their information in rewritable or read-only mode at bit level according to their own wishes, but can also work together with existing encryption techniques, which can further meet customized security requirements.

Except for spin-torque[1] driven race-track memory [28,29], magnetic domain-wall memories with non-volatility and high design flexibility have attracted more interest, with remarkable achievements in magnetic field-driven logic [30], SOT-driven magnetic logic [17,28] and neuromorphic computing [31,32]. The reciprocal motion of the current-driven domain-wall in this work could also be utilized for spin logic and neuromorphic computing, for example, through series and parallel connecting devices with materials of opposite spin Hall angle.

## CONCLUSION

In conclusion, we experimentally demonstrate wafer-scale electrically function-switchable domain-wall memory arrays by designing U-shaped domain-wall memory composed of Pt/Co/Ru/AlO_x_. The capped AlO_x_ layer can effectively reduce the critical current of SOT-driven domain-wall motion in the curved section of the U-shaped device by its thickness. With a strong DMI, the rewritable function can be realized by field-free SOT-induced domain-wall reciprocating motion with a small driving current density of (2.5 ± 0.2) × 10^6^ A/cm^2^. When the injected current is increased over a critical value, the domain-wall is annihilated at the edge of the ferromagnetic layer, inducing the stored information conversion from a rewritable to read-only state at bit level. The electrically function-switchable memory not only provides more versatile memory, but also paves the way for developing spin logic and neuromorphic computing.

## MATERIALS AND METHODS

### Thin film preparation

The films were deposited on Si/SiO_2_ substrate by magnetron sputtering at room temperature. DC magnetron sputtering was used to deposit the Ta, Pt, Co and Ru layers. AC magnetron sputtering was used to deposit the AlO_x_ layer. The base pressure of the chamber was less than 2.0 × 10^−6^ Pa. The pressure of the chamber was 1.06 × 10^−1^ Pa for Ta, Pt, Co and Ru, and 2.67 × 10^−1^ Pa for AlO_x_ during deposition, respectively. The deposition rates for Ta, Pt, Co, Ru and AlO_x_ layers were controlled at 0.022, 0.0240, 0.0124, 0.017 and 0.002 nm/s, respectively.

### Device fabrication

For a single device, UV lithography and magnetron sputtering deposition were used twice. First, the Ta (1 nm)/Pt (4 nm)/Co (0.85 nm)/Ru (1.2 nm) film was patterned into U-shaped devices with a channel width of 16 μm and inner diameter of 30 μm. Then, using photolithography engraving and magnetron sputtering deposition, an AlO_x_ layer with a thickness of 0.5 nm was grown in the curved section. Finally, argon ion beam etching was used to make trenches with a width of 5 μm in both straight sections, where Co/Ru bilayers were precisely etched away.

For the wafer-scale domain-wall memory arrays, every U-shaped device has a channel width of 10 μm and an inner diameter of 20 μm. The additional process of lift-off is needed to make the row electrodes (Ta (10 nm)/Au (50 nm)) and column electrodes (Ta (10 nm)/Au (150 nm)). To prevent electrical contact between the two layers of electrodes, a layer of SiO_x_ with a thickness of 100 nm is deposited by plasma-enhanced chemical vapor deposition (PECVD) to separate them.

### Measurement and characterization

The current-induced magnetization switching and anomalous Hall effect measurements were carried out at room temperature with a Keithley 2602B as the current source and Keithley 2182 as the nanovoltmeter. The Kerr imaging measurements were carried out using a NanoMOKE3 microscope.

## Supplementary Material

nwad093_Supplemental_File
