# Supplementary material for: Electrically function-switchable magnetic domain-wall memory
Source: Natl Sci Rev. 2023 Apr 10;10(10):nwad093. doi: 10.1093/nsr/nwad093 (PMC10476893; doi:10.1093/nsr/nwad093)
Supplement: nwad093_Supplemental_File [file nwad093_supplemental_file.docx]

SUPPLEMENTARY DATA

**Electrically switchable magnetic domain-wall memory**

Yu Sheng^1^, Weiyang Wang^1, 2^, Yongcheng Deng^1^, Yang Ji^1, 2^, Houzhi Zheng^1, 2^, Kaiyou Wang^1, 2*^

1 State Key Laboratory of Superlattices and Microstructures, Institute of Semiconductors, Chinese Academy of Sciences, Beijing, China.

2 College of Materials Science and Opto-Electronic Technology, University of Chinese Academy of Sciences, Beijing, China.

^*^Corresponding e-mail: [kywang@semi.ac.cn](mailto:kywang@semi.ac.cn)

**Contents**

S1. Magnetic properties and current-induced magnetization switching of the symmetric U-shaped Pt(4 nm)/Co(0.5 nm)/Pt(1.2 nm) device

S2. Endurance measurements of Pt(4 nm)/Co(0.5 nm)/Ru(1.2 nm) U-shaped devices

S3. Switching probability of the function switchable device from rewritable to read-only function

S4. Switching the device by short current pulses

**S1. Magnetic properties and current-induced magnetization switching of the symmetric U-shaped Pt(4 nm)/Co(0.5 nm)/Pt(1.2 nm) device**

In order to reveal the importance of Dzyaloshinskii−Moriya interaction (DMI) in current-induced magnetization switching in absence of magnetic field, through SOTs-driven domain wall motion, we fabricated the U-shaped device as reference based on Pt/Co/Pt stacks, where the DMI is very small due to the symmetric structure^1^.


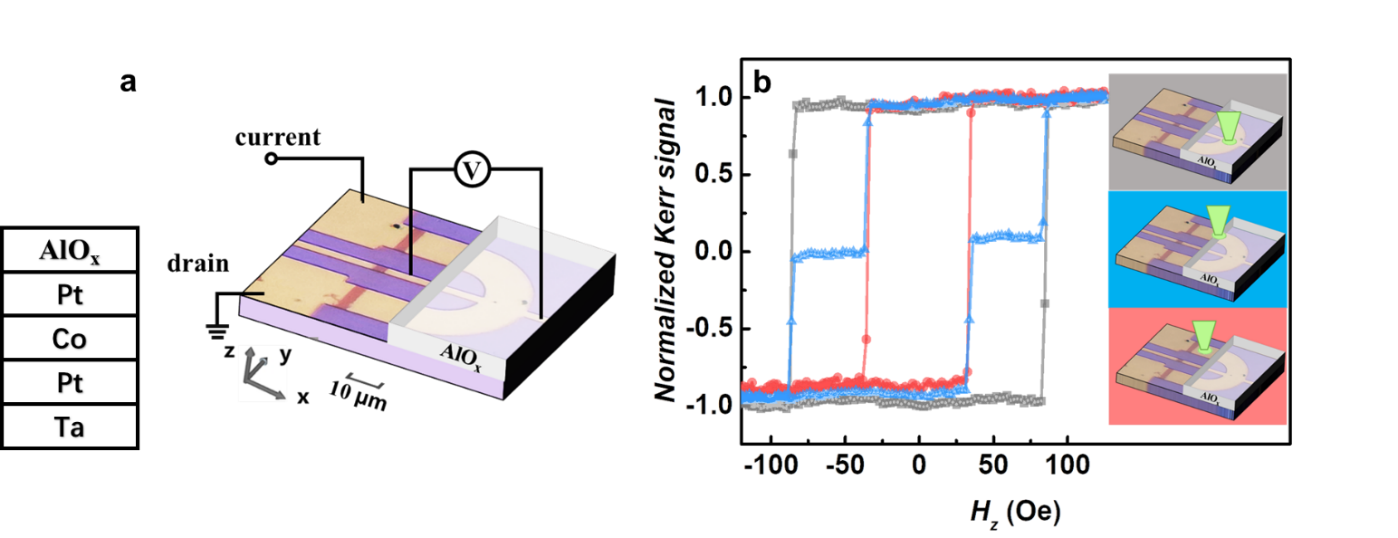


**Figure S1.** (a) Optical image of the U-shaped device and anomalous Hall effect measurement configuration for symmetric Pt/Co/Pt stacks with definition of x–y–z coordinates. The Co/Ru bilayer at both ends of the U-shaped region (red-brown stripes) are etched away by argon ion beam. (b) Out-of-plane hysteresis loops, obtained by a polar magneto-optical Kerr effect (pMOKE) microscope, of the U-shaped device in the Pt/Co/Pt region (red), Pt/Co/Ru/ AlO_x_ region (grey) and at the boundary (blue), as the inserts shown. The green cone in the inserting image represents the detecting laser from the pMOKE microscope.

As shown in Fig. S1, except for using the symmetric Pt/Co/Pt stacks, the U-shaped device structure is the same to that in Fig. 1a. The AlO_x_ capped layer was deposited on the curved region to locally weak the perpendicular magnetic anisotropy, creating a potential step at the boundary between the two regions (Fig. S1a). Magnetic hysteresis loops were measured using a polar magneto-optical Kerr effect (pMOKE) microscope, with a focused laser spot of 2 µm positioned at the straight section, curved section and the boundary between them (Fig. S1b). The switching fields in curved section and straight section are 35 Oe and 86 Oe, respectively, confirming the role of AlO_x_ capped layer. Double-step switching of the hysteresis loop at the boundary reveals the domain-wall can be pinned at the boundary (Fig. S1b).


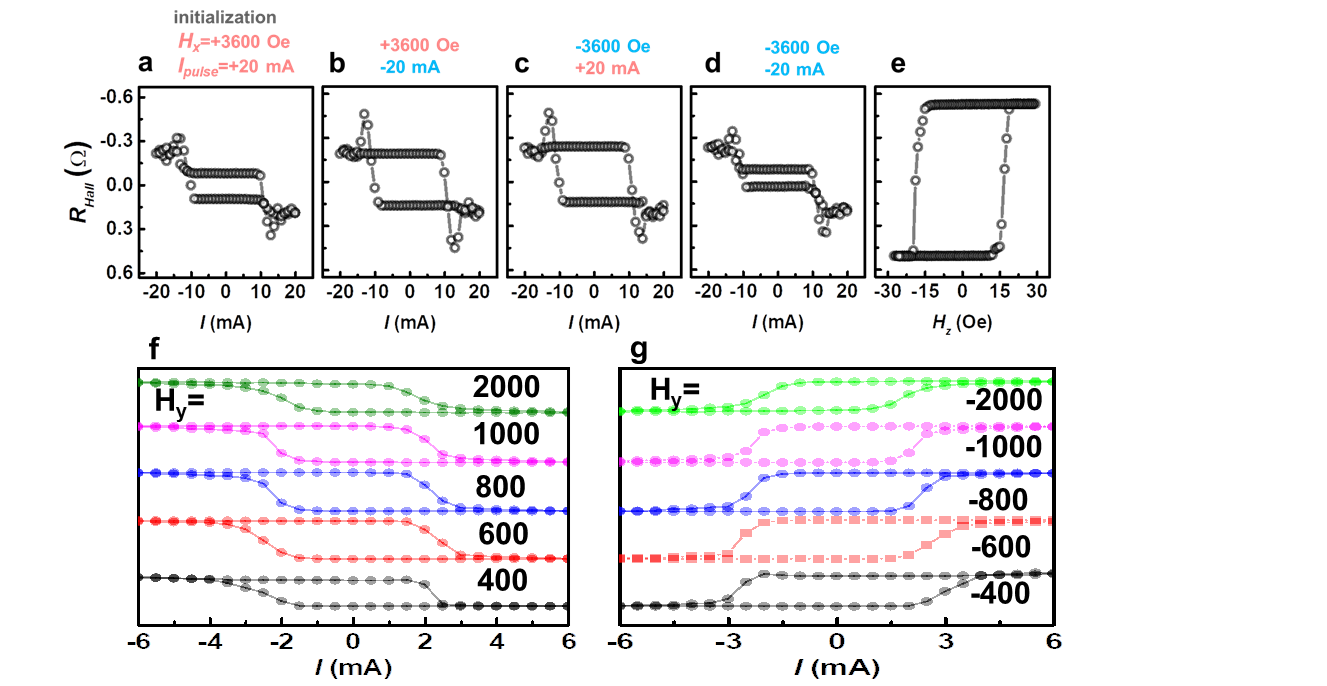


**Figure S2.** (a-d) Spin-orbit torques induced magnetization switching of perpendicular magnetization in the curved section (AlO_x_ capped) for four types of initializations: *H_x_*=±3600 Oe, *I_pulse_*=±20 mA. The pulse sequences consist of strings of pulses, each lasting 10 ms, with a scanning magnitude ranging from a maximum of *I_pulse_*=±20 mA. After each pulse is applied, all *R_Hall_* data points are measured 100 ms later with a detecting current of 0.1 mA. (e) Out-of-plane magnetic field induced switching loop of the perpendicular magnetization in the curved section. (f-g) Magnetization switching by current under positive (f) and negative (g) in-plane magnetic fields. All the data are measured for the device with symmetric Pt(4 nm)/Co(0.5 nm)/Pt(1.2 nm) structure.

We then investigated the current-driven domain-wall motion in the symmetric U-shaped device. In Fig. S2a-d, four types of initializations with *H_x_*=±3600 Oe and *I_pulse_*=±20 mA were performed to create opposite domain in two straight sections, and then we measured the anomalous Hall resistance vs. the electrical current pulses after each initialization. Similar clockwise-like switching loops were observed in response to current pulse for all four types of initializations, where the switching magnitudes of *R_Hall_* after initializations of *H_x_*=3600 Oe & *I_x_*=22 mA, *H_x_*=3600 Oe & *I_x_*=-22 mA, *H_x_*=-3600 Oe & *I_x_*=22 mA and *H_x_*=-3600 Oe & *I_x_*=-22 mA are 0.17 Ω, 0.35 Ω, 0.38 Ω and 0.11 Ω, respectively, with the switching amplitudes of *R_Hall_* being much smaller than the switching amplitude caused by perpendicular magnetic field (1.04 Ω) (Fig. S2e).

There are two main roles of the top layer in SOTs-driven domain-wall motion in Pt/Co/X structure, where X indicates a kind of normal metal. One is influencing the net spin current by spin reflecting, spin absorbing and spin Hall effect, the other is affecting the amplitude of DMI. The DMI can decide whether the domain wall can be driven by current under zero magnetic field, when the net spin current is not zero. For the symmetric Pt(4 nm)/Co(0.5 nm)/Pt(1.2 nm) but with different thickness of Pt, we experimentally demonstrate that the net spin current is large enough to switch the magnetization of Pt/Co/Pt device with the assistance of an in-plane magnetic field (Fig. S2f-g). However, with the current far larger than the field-assistance-critical switching current, the electric current itself cannot drive the domain-wall motion to realize rewritable function. The DMI of Pt/Co/Ru (2.3~2.66 mJ/m^2^) is much larger than Pt/Co/Pt (0.1~0.8 mJ/m^2^) as the asymmetric structure. Thus, the rewriteable function can only be realized in the asymmetric structure device with larger DMI, confirming the importance of the DMI for the SOTs-driven domain-wall memory.

**S2. Endurance measurements of Pt(4 nm)/Co(0.5 nm)/Ru(1.2 nm) U-shaped devices**


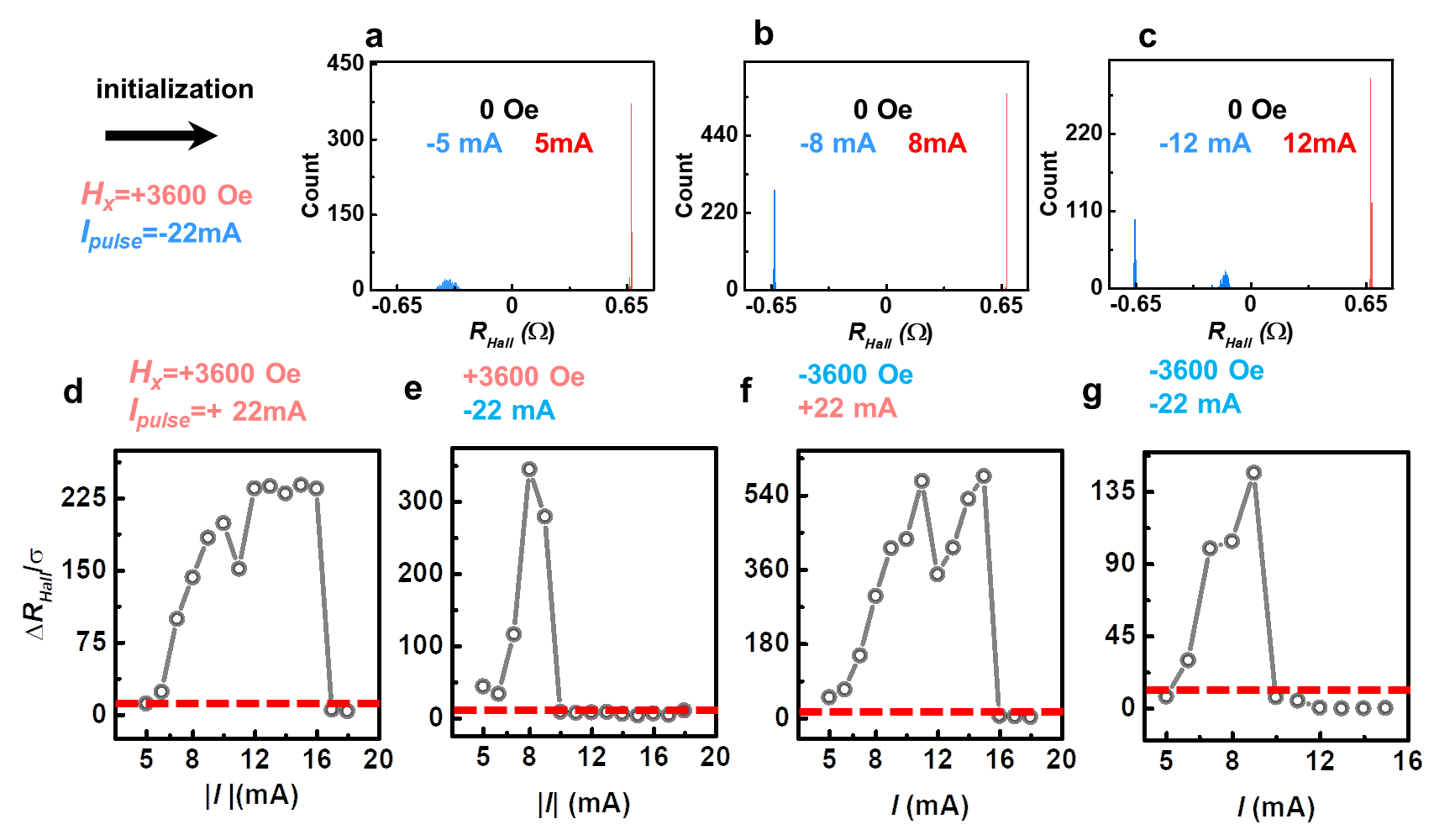


**Figure S3.** (a-c) Field-free deterministic spin–orbit torques switching for initialization of *H_x_*=3600 Oe & *I_x_*=-22 mA. The alternately positive and negative current pulse sequences of 1000 cycles are applied with duration of 10 ms and amplitude of 5 mA, 8 mA and 12 mA for (a), (b) and (c) respectively. Red line indicates *R_Hall_* statistics with positive current pulses, while blue line indicates *R_Hall_* statistics with negative current pulses. (d-g) The amplitude of the current pulse |*I*| versus Δ*R_Hall_* /σ, which is the average change in resistance from the low- to high-state (Δ*R_Hall_*) divided by averaged standard deviation of two states (σ), for four types of initializations of *H_x_*=3600 Oe & *I_x_*=22 mA (d), *H_x_*=3600 Oe & *I_x_*=-22 mA (e), *H_x_*=-3600 Oe & *I_x_*=22 mA (f) and *H_x_*=-3600 Oe & *I_x_*=-22 mA (g). Red dashed line in (d)-(g) indicates the line where Δ*R_Hall_* /σ equals to 12.

It is extremely important for the rewritable function to have high endurance and wide operation window for applications. To check the endurance and operation window, we perform the endurance measurements under different amplitudes of alternately positive and negative current pulses for 1000 cycles. As shown in Fig. S3a, after initialization of *H_x_*=3600 Oe, *I_pulse_*=-22 mA, a current pulse of -5 mA prefers down-magnetization with *R_Hall_*=-0.37 Ω, and a current pulse of 5 mA prefers up-magnetization with *R_Hall_* =0.67 Ω. The ideal case is that both up- and down-magnetization states show the magnitude of 0.67 Ω. The much smaller magnitude at -5 mA indicates an incompletely switching. As shown in Fig. S3b, a current pulse of -8 mA prefers down-magnetization with *R_Hall_*=-0.64 Ω, and a current pulse of +8 mA prefers up-magnetization with *R_Hall_* =0.67 Ω. Thus, the added-up amplitude of +8 mA pulse induced magnetization switching is 1.31 Ω, which is comparable to the amplitude of field-induced switching (1.34 Ω). However, further increasing the current amplitude to 12 mA, except for the two main peaks at 0.67 Ω, another resistance level at -0.14 Ω can also be observed, indicating a multi-domain in the cross area. Fig. S3d-g show the ΔR/σ versus current amplitude |*I*| under four types of initializations, and the operation windows ensuring that ΔR/σ larger than 12 are 10 mA, 4 mA, 10 mA and 4 mA for *H_x_*=3600 Oe & *I_x_*=22 mA, *H_x_*=3600 Oe & *I_x_*=-22 mA, *H_x_*=-3600 Oe & *I_x_*=22 mA and *H_x_*=-3600 Oe & *I_x_*=-22 mA, respectively. Therefore, we achieved a wide electric operation window of 10 mA for rewritable magnetic domain-wall memory, which meets the requirements for future applications.

**S3. Switching probability of the device from rewritable to read-only function**

The switching probability of the device from rewritable to read-only function was measured after initialization of *H_x_*=3600 Oe & *I_x_*=22 mA. First, ten cycles of alternating current pulses with amplitude of 9 mA and duration of 10 ms were applied to confirm the realization of rewritable function. Then, a current pulse *I* was applied to switch the device to read-only function, and followed by a set of pulses (-9mA and +9 mA) to detect whether the switching was successful or not. After each pulse, a small current of 0.1 mA was applied to detect the anomalous Hall resistance. To investigate the switching probability, we counted the number (n) of successful switching to read-only function in 10000 pulses, and took n/10000 as the switching probability. The switching probability for the measured current pulse ranged from 14.5 to 22 mA is shown in Fig. 3d in the main manuscript.

**S4. Switching the device by short current pulses**

Short pulses are applied to the device to test the time response. Anomalous Hall resistance versus current pulses of 22 mA with duration of 10 μs and 1 μs is shown in Fig. S4 a and b, respectively. The device can be fully switched with the duration of a current pulse of 10 μs and only partly switched with 1 μs, which is due to large size of the device structure in our work. The curved region of U-shaped device has a channel width of 16 μm and inner diameter of 30 μm. The DW need to pass through at least the whole curved region to achieve the full switching, thus the DW speed can be estimated to be 14.5m/s. The moving speed of the domain wall driven by SOTs in Pt/Co system is in tens of m/s[2]–[5] with current density of 10^6^A/cm^2^. The nanoscale magnetic domain wall device has been reported to response on the order of ns [6]. Thus, the function speed of our device can be operated in ns regime with shrinking the device into nm-scale.


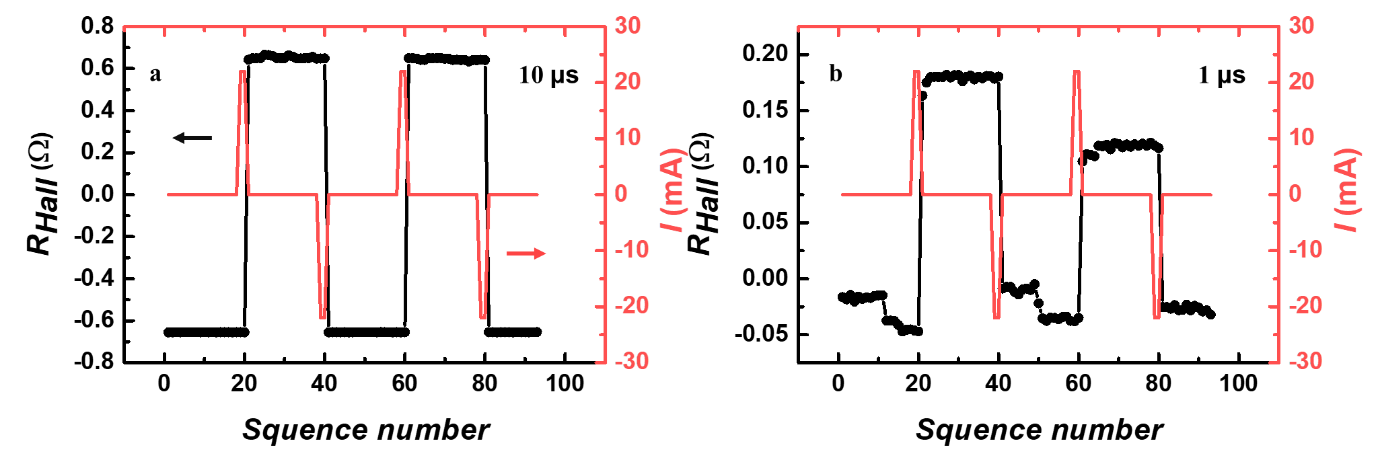


**Figure S4.** The black curve indicates the Hall resistance, and the red curve in a and b indicates the current pulse with duration of 10 μs and 1 μs respectively. The device was initialized by H_X_=3600 Oe and I_x_=-22 mA with duration of 10ms before measurement.

References and Notes.

[1] Hrabec A, Porter N A and Wells A *et al.* Measuring and tailoring the Dzyaloshinskii-Moriya interaction in perpendicularly magnetized thin films. *Phys. Rev. B* 2014; **90**: 020402.

[2] Guan Y, Zhou X, Ma T *et al.* Increased Efficiency of Current-Induced Motion of Chiral Domain Walls by Interface Engineering *Adv. Mater.* 2021; **2007991**: 1–9.

[3] Rojas-s J. Very large domain wall velocities in Pt/Co/GdOx and Pt/Co/Gd trilayers with Dzyaloshinskii-Moriya interaction *EPL.* 2016; **113**: 67001.

[4] Yun J, Li D, Cui B *et al.* Current induced domain wall motion and tilting in Pt/Co/Ta structures with perpendicular magnetic anisotropy in the presence of the Dyzaloshinskii – Moriya interaction *D Appl. phys.* 2018; **51**: 155001.

[5] Emori S, Bauer U, Ahn S-M *et al.* Current-driven dynamics of chiral ferromagnetic domain walls *Nat. Mater.* 2013; **12**: 611–616.

[6] Raymenants E, Bultynck O and Wan D et al. Nanoscale domain wall devices with magnetic tunnel junction read and write. *Nat. Electron.* 2021; **4**: 392–8.
